# Energy Drinks and Myocardial Infarction

**DOI:** 10.7759/cureus.2658

**Published:** 2018-05-21

**Authors:** Muhammad Wajih Ullah, Sunita Lakhani, Wardah Siddiq, Arshi Handa, Yamini Kahlon, Tariq Siddiqui

**Affiliations:** 1 Cardiology, Mayo Clinic, Rochester, USA; 2 Internal Medicine, Liaquat University of Medical and Health Sciences Hospital Jamshoro Sindh Pakistan., Jamshoro, PAK; 3 Internal Medicine, Beth Israel Deaconess Medical Center/ Harvard Medical College, Boston, USA; 4 Internal Medicine, Poznan University of Medical Sciences, Poznań, POL; 5 Internal Medicine, American University of Antigua, New york, USA; 6 Internal Medicine, Maharashtra Institute of Medical Education & Research, Talegaon, IND

**Keywords:** myocardial infarction, energy drink

## Abstract

The popularity and use of energy drinks have accelerated over the past decade and are a health concern worldwide. The key ingredients of energy drinks include caffeine, guarana, taurine, ginseng, and sugar. Most of the well-known side effects due to consumption of energy drinks include tachycardia, headache, anxiety, and palpitations and are frequently attributed to caffeine. Recently, a few cases of life-threatening cardiovascular events in individuals who overdosed massive quantities of caffeinated energy drinks have been reported. In this case report, we are documenting a case of myocardial infarction in a 25-year-old man who presented to the emergency department with chest pain. The patient had been consuming massive quantities of caffeinated energy drinks daily for the past week. This case report and the few previously documented studies support a possible connection between caffeinated energy drinks and myocardial infarction. The purpose of this case report is to promote awareness in the general population and the medical staff about cardiac mortality due to overdosing of massive quantities of caffeinated energy drinks.

## Introduction

Over the past decade, the popularity of energy drinks has accelerated, particularly among adolescents. In 2006, more than 30% adolescents reported consuming energy drinks regularly [1]. The widespread use of energy drinks has brought great health concerns worldwide [2-3]. Several studies postulated caffeinated energy drinks to have a direct link to cardiac mortality [4-5]. We report a case of myocardial infarction in a 25-year-old man who ingested a massive quantity of caffeinated energy drinks for the past week leading to the event.

## Case presentation

An otherwise healthy 25-year-old man presented to the emergency department with a substernal chest pain for an hour accompanied by shortness of breath, nausea, and vomiting. The chest pain was sudden in onset, 8/10 in intensity, and radiating to his right arm. The chest pain was slightly relieved on lying flat and aggravated by walking. He had no associated symptoms such as fever, cough, runny nose, or rash. He did not have any antecedent infection.

Patient’s past medical, surgical and family history was unremarkable, and he had no modifiable or non-modifiable cardiovascular risk factors. He had no known allergic reaction to food or drugs. He was a nonsmoker and did not use any illicit drugs. A comprehensive history revealed a daily intake of seven to nine cans of caffeinated energy drinks for the past week. The patient reported significant improvement in his chest pain after receiving sublingual nitroglycerin and diamorphine intravenously.

His vital signs on examination were (1) Temperature: afebrile, (2) Blood Pressure: 155/95 mmHg in his right arm and 150/90 mm Hg in his left arm, (3) Respiratory Rate: 25 breaths/min, d-Heart Rate: 110 beats/min. Pulse oximetry showed 98% oxygen saturation on room air. Cardiac examination revealed S4 on auscultation of the chest. On palpation of the chest, there was no point tenderness. Rest of the systemic examination was unremarkable.

**Figure 1:**
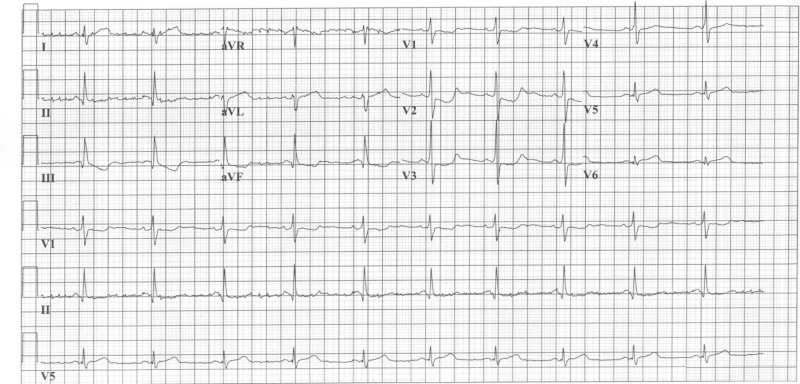
EKG EKG showing ST depression in precordial leads V2-V6

The initial electrocardiogram (EKG) on admission (Figure *1*) showed sinus rhythm with ST depression in precordial leads V2-V6. Chest X-ray was insignificant with no signs of pulmonary congestion. Laboratory findings were as follow: (1) an elevated level, 32.22 µg/ml, of 12-h troponin I (normal range <0.07); confirming definite acute coronary syndrome. (2) d-Dimers were 380 ng/ml (normal range <500). (3) Thrombophilia and drug screen were negative. After appropriate initial resuscitation for the coronary syndrome, the patient was transferred to the catheterization laboratory for the percutaneous coronary intervention.

Coronary angiography showed normal coronary arteries. Transthoracic echocardiography (TTE) showed ejection fraction of 60% with left ventricular diameters within normal limits. The patient was discharged after five-day observation period and was counseled to limit/stop the ingestion of energy drinks. He was discharged with aspirin, spironolactone, perindopril, and atenolol.

On the post-hospital follow-up visit, the patient had completely stopped consuming energy drinks and was symptom-free. TEE at this visit revealed ejection fraction of 62% with preserved global left ventricular function.

## Discussion

Recently, the recreational use and popularity of the commercial energy drinks have increased worldwide. The growth in consumption of energy drink has led to an increase in the number of reports about its life-threatening toxicity [6-7]. Energy drinks contain many ingredients, but the key ingredients of energy drinks include caffeine, guarana, taurine, ginseng, and sugar. Most of these ingredients except caffeine are in subtherapeutic doses in the energy drinks and therefore, they bear no hazards [8]. The most studied substance in the energy drinks is caffeine. The lethal oral caffeine dose is 10 grams [9]. One can of a commercial energy drink contains 40–200 mg of caffeine. The maximum recommended daily dose of caffeine for an adult is 400 mg [9]. The therapeutic index of caffeine is remarkably high and side effects such as an increase in blood pressure, nausea, and palpitations are seen with mild caffeine intoxication [10]. Massive overdoses of caffeine may result in life-threatening toxicity [11].

A few studies have been documented in the past which linked excessive caffeine intake with myocardial infarction. In 1997, a case of a young woman was reported to suffer from myocardial infarction due to the massive overdose, 20 grams, of caffeine [12]. Another study in 2009 reported a cardiac arrest in a young man after he consumed excessive amounts of caffeinated energy drinks throughout the day. The authors of the mentioned case postulated that a combination of caffeinated energy drinks and strenuous physical activity caused coronary vasospasm leading to the cardiac arrest [5]. Further, a study in 2011 documented energy drinks increase platelet aggregation and cause endothelial dysfunction which potentially can trigger myocardial infarction [10].

In our patient, his angiography was normal and his urine drug screen was negative for any drugs excluding vasospastic causes of myocardial infarction such as Prinzmetal’s angina, and cocaine-induced myocardial vasospasm respectively. His TEE revealed normal left ventricular wall function which excluded hypertrophic obstructive cardiomyopathy or Takotsubo syndrome. The case illustrated in our case report was electrocardiographically and biochemically proven myocardial infarction. Our patient started consuming seven to nine cans of energy drinks daily one week prior to the event. Since each can of energy drink contains approximately 200 milligrams of caffeine, our patient was consuming almost 1400-1800 milligrams of caffeine which is much higher than the standard recommended dose of 400 milligrams per day (4x overdose). We suspect that the myocardial infarction our patient experienced was secondary to his overdosing of caffeinated energy drinks in the days preceding his admission.

## Conclusions

Although there is no literature proving energy drinks as a cause of cardiac mortality, in the light of recent increase in consumption of caffeinated energy drinks, it is very important to understand the negative side effects energy drinks have in consumers. In the context of this case report, previously documented case reports, and in the presence of reasonable pharmacological mechanisms, we believe that the adverse events of these caffeinated energy drinks should be highlighted. We encourage clinicians to screen their patients for energy drink use (or abuse) and provide patients the better understanding of the risks due to ingestion of massive doses of the energy drinks.
